# The schemes, mechanisms and molecular pathway changes of Tumor Treating Fields (TTFields) alone or in combination with radiotherapy and chemotherapy

**DOI:** 10.1038/s41420-022-01206-y

**Published:** 2022-10-11

**Authors:** Guilong Tanzhu, Liu Chen, Gang Xiao, Wen Shi, Haiqin Peng, Dikang Chen, Rongrong Zhou

**Affiliations:** 1 Department of Oncology, Xiangya Hospital, Central South University, 410008 Changsha, China; 2 Hunan An Tai Kang Cheng Biotechnology Co., Ltd, Changsha, China; 3 National Clinical Research Center for Geriatric Disorders, Xiangya Hospital, Central South University, Changsha, 410008 Hunan Province P.R. China; 4 Xiangya Lung Cancer Center, Xiangya Hospital, Central South University, 410008 Changsha, China

**Keywords:** Cancer therapy, CNS cancer, Non-small-cell lung cancer, Autophagy, Apoptosis

## Abstract

Tumor Treating Fields (TTFields) is a physical therapy that uses moderate frequency (100–300 kHz) and low-intensity (1–3 V/cm) alternating electric fields to inhibit tumors. Currently, the Food and Drug Administration approves TTFields for treating recurrent or newly diagnosed glioblastoma (GBM) and malignant pleural mesothelioma (MPM). The classical mechanism of TTFields is mitotic inhibition by hindering the formation of tubulin and spindle. In addition, TTFields inhibits cell proliferation, invasion, migration and induces cell death, such as apoptosis, autophagy, pyroptosis, and cell cycle arrest. Meanwhile, it regulates immune function and changes the permeability of the nuclear membrane, cell membrane, and blood-brain barrier. Based on the current researches on TTFields in various tumors, this review comprehensively summarizes the in-vitro effects, changes in pathways and molecules corresponding to relevant parameters of TTFields (frequency, intensity, and duration). In addition, radiotherapy and chemotherapy are common tumor treatments. Thus, we also pay attention to the sequence and dose when TTFields combined with radiotherapy or chemotherapy. TTFields has inhibitory effects in a variety of tumors. The study of TTFields mechanism is conducive to subsequent research. How to combine common tumor therapy such as radiotherapy and chemotherapy to obtain the maximum benefit is also a problem that’s worthy of our attention.

## Facts

- TTFields inhibits the growth of various tumors, such as GBM, lung cancer, malignant pleural mesothelioma, liver cancer, ovarian cancer, and pancreatic cancer.
- The inhibition of cell proliferation, migration, and invasion by TTFields depends on frequency, intensity, duration, and direction.
- TTFields causes multiple death modes, such as apoptosis, autophagy, immunogenic cell death, and pyroptosis.
- TTFields combined with radiotherapy or chemotherapy generally exerts a synergistic effect.
- TTFields alone or combined with radiotherapy and chemotherapy affects the Fanconi Anemia-BRCA, cGAS-STING, NF-κB, MAPK, and PI3K/AKT signaling pathways.

## Open questions

- Could TTFields lead to a new mode of cell death?
- Which regimen can cause maximum tumor suppression when TTFields alone or combined with radiotherapy and chemotherapy?
- Can bioinformatics analysis such as single-cell sequence reveal more mechanisms for TTFields?
- What other signaling pathways can TTFields affect?

## Introduction

TTFields is a physical tumor therapy that bases on medium frequency (100-300 kHz) and low-intensity (1–3 V/cm) alternating electric fields. In vitro/vivo experiments and clinical trials have shown that TTFields inhibits the growth of various tumors (such as GBM [1–8], lung cancer, malignant pleural mesothelioma [9–13], liver cancer [14, 15], ovarian cancer [16, 17] and pancreatic cancer [18, 19]), and prolongs survival. Furthermore, combined with radiotherapy [20–25], chemotherapy [22, 26–38], and other treatments, TTFields obtains better therapeutic effects. As a non-invasive physical therapy, TTFields has mild adverse reactions, mostly grade 1–2 cutaneous adverse reactions such as mild to moderate rash under the electrodes [3, 6], erythema, dermatitis, pruritus [2, 4, 9, 10, 14, 15, 19, 39, 40], erosions [24, 41, 42], with no or minimal grade 3 skin adverse events [2, 10, 14]. Reassuringly, these symptoms improve with steroid treatment, electrode replacement, or temporary cessation of TTFields [2, 9, 10, 19].

The classic mechanism of TTFields is to interfere with the mitosis of tumor cells, but it has little effect on normal cells [43]. Moreover, follow-up studies have demonstrated that TTFields induces various functions such as cell death, changes in cell membrane permeability, and immune regulation. Although previous researches have summarized the effects of TTFields on gliomas, GBM, and other tumors, this review focuses on in-vitro studies of various tumors. It comprehensively lists the experimental parameters, making it more convenient and clearer to update the research status of TTFields. In addition, we firstly summarized the comprehensive changes in molecular pathways after TTFields.

### The parameters of TTFields: inhibit proliferation, migration, and invasion of tumor cell

The inhibition of proliferation, migration, and invasion by TTFields depends on frequency [41, 44, 45], intensity [1, 20, 23, 28, 30, 31, 45, 46], duration [23, 47–49], direction [41, 50, 51], and cell volume [49].

The commonly used frequency of TTFields on tumor cells is 100-200 kHz. In contrast, a few tumor cells (such as MZ-54, DAOY, and some primary cells) are out of the range [41, 44, 45]. Non-small cell lung cancer, cervical cancer, breast cancer, pancreatic cancer, and osteosarcoma have an optimal frequency of 150 kHz while ovarian cancer, glioma, GBM, or GBM-like stem cells are general at 200 kHz [49]. Malignant pleural mesothelioma is mostly inhibited at 150 or 200 kHz [28, 48]. However, the inappropriate frequency may promote cell growth. The higher frequency could weaken inhibitory effect, but the mechanism has not been studied yet [20, 23, 28, 45, 52]. Meanwhile, tumor cell growth is favored at fragile intensity and non-optimal frequency of TTFields [50]. Giladi et al. [48] report that the optimal inhibitory frequency is related to the doubling time of tumor cells. In addition, the optimal frequency remains consistent in different intensities [46].

The inhibitory effect of TTFields is time-dependent. Generally, 48–72 hours [23, 47–49], and the duration in a part of studies is ≤24 hours [1, 20, 31, 41, 51, 53] or >100 hours [35, 36, 48, 54–56]. The dependence of duration on tumor cell suppression is significantly reduced when the duration exceeds 6 hours/day. Cytostatic effect appears indistinguishable for the same duration, no matter TTFields administered continuously or dividedly [57].

Commonly applied intensity ranges 1–2 V/cm, mostly 1.75 V/cm, with a few studies in relatively low or high intensity (0.6 V/cm or >4 V/cm) [36, 37, 51]. Generally, the inhibition of TTFields is intensity-dependent [1, 20, 23, 28, 30, 31, 45, 46]. TTFields have a certain intensity threshold for tumor inhibition. When the intensity is <0.7 V/cm, no significant reduction in tumor volume is observed [34, 35].

Different TTFields directions have different inhibition effects. Parallel or perpendicular application of TTFields significantly reduces scratching speed, migration distance and direction, and cell polarization. Moreover, compared with the parallel application of TTFields, the vertical one has a more significant effect on the migration velocity [51]. However, some studies show that TTFields functionates when its direction is parallel to the spindle [50]. Increasing TTFields directions enhances inhibitory efficiency [41]. The inhibitory effect of TTFields on cells is also related to cell size [49].

Researches at present mainly focus on tumor cell lines, with a few studies on primary GBM cells [36, 41, 54]. Few studies have focused on drug-resistant strains: cell lines of pancreatic cancer [29, 50], breast cancer [27, 33] and GBM-like stem cells [54], ovarian cancer [27]. Their sensitivity frequencies are consistent with standard tumor cell lines. In addition, a few studies have reported the effect of TTFields on animal cell lines [1, 27, 34, 35, 53, 58]. The TTFields parameters of tumor inhibition are shown in Table 1.

**Table 1:** Overview of the frequency, intensity, duration, and effect of TTFields alone on various tumor cells.

Cancer type	Cell	Species	Frequency (kHz)	Intensity (V/cm)	Time and effect	Reference	Device	Note
Breast cancer	MCF-7, MCF-7/Mx, MDA-MB-231, MDA-MB-231/Dox	Human	150	1.75	72 h: wild-type and ABC transporters-expressing resistant cells proliferation ↓	[27]	The inovitro^TM^ system	MCF-7/Mx has ABC transporter
	MCF-7, MDA-MB-231		150	0.63,1.1,1.75,4	24 h or 72 h: proliferation and clonal formation ↓ , Intensity dependent	[1, 31, 36, 48]	The inovitro^TM^ system	Doubling time :29.3 h
Cervical cancer	HeLa	Human	150	1.75	Apoptosis ↑	[38, 48]	The inovitro^TM^ system	Doubling time :24 h
Colon cancer	HCT116	Human	150	1	24 h:TP53 dependence, apoptosis↑	[66]	–	–
	CT-26	Mouse	200	1.75	24h-72h: apoptosis ↑	[52]	–	–
Ependymoma	DKFZ-EPN1, BXD-1425EPN	Human	100, 200	1.75	72 h: Cell count↓	[45]	The inovitro^TM^ system	–
Glioblastoma	Primary cells	Human	150-220	1–2.2	24 h: cell count = , 48 h: cell count ↓ . Intensity dependency, TEFT-random ≥ TEFT-fixed	[41]	TEFTS, CL-301A	–
	GaMG, U-343 MG, U-138 MG, KNS42, GIN-31, LN-229, LN-18		200	0.6, 1.7,1.75	24 h: Invasion↓ or 72 h: proliferation↓	[1, 45, 49, 51]	The inovitro^TM^ system	–
	MZ-54		250	1.48	72 h: cell count↓	[44]	The inovitro^TM^ system	–
	Primary cells GBM2, GBM39		200	4	100 h: GBM39 proliferation ↓ , 150-200 h: GBM2 proliferation↓	[36]	The inovitro^TM^ system	–
	U251		200	1.48	–	[44]	The inovitro^TM^ system	–
	U-87 MG, U-118 MG, A-172		200	0.6,1.7,1.75	24 h: migration and invasion↓ or 72 h: proliferation and clonal formation↓	[48, 49, 51, 58]	The inovitro^TM^ system	Doubling time :34 h
	U87-MG, U-373 MG, 528NS, 83NS		150	0.9	48 h or 72 h: proliferation, clonal formation, migration, invasion and EMT-associated protein expression ↓ , apoptosis ↑	[47, 55, 56]	Self-made	–
	U87-MG		200	4	24 h: apoptosis = , 240 h: proliferation↓	[36, 63]	The inovitro^TM^ system	–
	patient-derived GBM stem-like cells (GSCs): TMZ resistant/sensitive		200	1	–	[54]	The inovitro^TM^ system	–
Glioma	U-118, U-87, LN-18, LN-229, T-325, ZH-161	Human	100	1.1,2	24 h: proliferation, invasion and migration↓	[1, 20]	–	–
					48–72 h: caspase-independence apoptosis ↑			
	U373		150	1.2	<24 hours, with time goes by, tumor cell apoptosis↑ but not in normal cell	[57]	–	–
	F98	Rat	200	1.1, 1.7, 1.75	24 h: Cell count↓ or 72 h: proliferation and clonal formation↓	[1, 57]	The inovitro^TM^ system	–
Liver cancer	HEPG2, Huh7	Human	150	1.75	24h-72h: Apoptosis ↑	[38, 52]	–	–
MPM	MSTO-211H, NCI-H2052	Human	150, 200	1–1.5,1.75	72 h: Proliferation and clonal formation ↓ . apoptosis ↑	[28, 48, 58]	The inovitro^TM^ system	Doubling time :18.9 h
Medulloblastoma	DAOY, UW228-3	Human	300, 100	1.75	72 h: Cell count↓	[45]	The inovitro^TM^ system	–
Melanoma	B16F10	Mouse	100	1.1 v/cm or peak Voltage:30 v	24 h: Cell count ↓ . Peak voltage-dependent manner	[1, 53]	Self-made or the inovitroTM system	–
Lung cancer	H157, H4006, A549, NCI-H1299, H1650, HTB-182, HCC827 (NSCLC)	Human	100, 150, 150/200, 100, 100	1.75	72 h: Proliferation and clonal formation↓	[25, 34, 48]	The inovitro^TM^ system	Doubling time :23.8 h
	LLC1, KLN205	Mouse	150	1.75	72 h: cell count↓	[34, 58]	The inovitro^TM^ system	–
	H520(Squamous cell lung cancer)	Human	150	1.75	24h-72h: apoptosis ↑	[52]	–	–
Osteosarcoma	U2OS, KHOS/NP	Human	150	1.5	48 h: cell count, migration and invasion↓	[81]	–	–
Ovarian cancer	A2780, OVCAR3, CAOV-3	Human	200	1.7,1.75, 4.6	72 h: proliferation ↓	[37, 46, 48]	The inovitro^TM^ system	Doubling Time :18.7 h
	MOSE-L	Mouse	200	1.75	24–72h: apoptosis ↑	[52]	–	–
	EmtR1	Hamster	150	1.75	72 h: wild-type and ABC transporters-expressing resistant cells ↓	[27]	The inovitro^TM^ system	EmtR1 cells ATP dependent MDR1 type drug resistance
Pancreatic cancer	CFPAC-1, HPAF-11, AsPC-1(Human), Pc-1.0 (hamster)	Human, hamster	150	1.75, 1.2, 2.9 ± 0.2	48 h or 72 h: proliferation and clonal formation ↓	[23, 35, 48, 58]	The inovitro^TM^ system or Self-made	Doubling Time :54 h
	BxPC–3, BxPC-3 cells BxGem cell, AsPC-1, non-malignant human hTERT-HPNE immortalized pancreatic duct cell line CRL-4032	Human		–	96 h: BxPC-3, BxGem, AsPC-1 cell proliferation ↓ , CRL-4032:no effect.	[50]	Self-made	150 kHz is the optimal frequency of BxPC-3 or BxGem AsPC-1, inhibiting cell proliferation and having no effect on CRL-4032
					144 h: apoptosis and necrosis=			

*↑* up-regulate, *↓* down-regulate, *=* unchanged.

*ABC transporters* ATP-binding cassette transporters, *EMT* epithelial-mesenchymal transition, *TMZ* temozolomide, *GBM* glioblastoma, *MDA-MB-231/Dox cells* doxorubicin resistant MDA-MB-231 cells, *EmtR1 cells* AA8 cells- Emetine-resistant sub-lines, *MCF-7/Mx* MCF-7 cells Mitoxantrone-resistant sub-lines, *BxGem cell* gemcitabine-resistant BxPC-3 cells.

### The different effects of TTFields on tumor cells and normal cells

The most classical mechanism of different effects of TTFields on tumor cells and normal cells bases on the difference in the biological behavior of two cells. Characterized by maintaining proliferative signals, evading growth inhibition, tumor cells have shorter doubling time and more vigorous mitosis than normal cells [59]. Meanwhile, inhibition by TTFields negatively correlates with doubling time of cells [48]. The different effects of TTFields on normal cells and tumor cells reflected in the following four aspects:

Cell proliferation and death. TTFields inhibits the proliferation of neural stem cells but not astrocytes [45]. Similarly, TTFields significantly suppresses tumor proliferation and induces apoptosis when applied to the skin or abdomen [50, 60] while the normal cells are unaffected [38, 50, 60, 61]. However, normal cells HaCaT proliferated slightly after TTFields [62].

DNA damage repair and cell cycle arrest. TTFields inhibits tumor cells and causes DNA damage [62] but does not cause DNA double-strand breaks and cell cycle arrest in normal cells [57, 61]. However, studies show that TTFields leads to G2/M arrest in IEC6 normal cells and tumor cells, but the increased degree varies with the duration of TTFields (0–24 hours/day) [57].

Duration. Within 12 hours treatment of TTFields, there is no significant change in IEC6 normal cells. Apoptosis cells slightly rise at 24 hours, but are far less than that of tumor cells [57]. When TTFields treats for 3–12 hours/day, the inhibition on normal cells and tumor cells is quite different. However, when the duration is longer than 24 hours/day, the degree of differential inhibition decreases [45].

Cell membrane permeability. TTFields increases the number and diameter of membrane pores in tumor cells but does not affect normal cell membranes [63].

### Apoptosis

TTFields alone or combined with hyperthermia or drugs such as Paclitaxel, Sorafenib, and MPS1-IN-3 (spindle assembly checkpoint inhibitor) increase apoptosis in glioma or GBM [26, 32, 45, 49, 55, 57, 64, 65]. In general, the portion of apoptosis cells varies among cell lines, is positively related to the intensity [20]. However, some studies indicate that TTFields does not increase apoptosis at higher field intensity and optimal inhibition frequency [20, 63]. Inhibition of autophagy leads to increased apoptosis and cell death [58].

TTFields alone or in combination with drugs (5-Fluorouracil, Paclitaxel) on various tumors such as ovarian cancer [37, 48], colon cancer [30, 52, 66], melanoma [53, 61], MPM [28], and breast cancer [33] also induces apoptosis. However, TTFields combined with the drugs does not necessarily and synergistically increase apoptosis. Thymidine attenuates TTFields-induced apoptosis in glioma cells [62]. TTFields restraints Osimertinib-induced apoptosis in lung adenocarcinoma cells and reduces the efficacy of Osimertinib [29].

### Autophagy

Aberrant mitosis, aneuploidy, and increased cellular granularity often induce prominent autophagy [67]. Time-lapse microscopy monitoring of mitotic index, mitotic duration, and intracellular autophagosome formation during TTFields demonstrates that TTFields induces autophagy due to abnormal mitosis and endoplasmic reticulum stress [58]. TTFields induces autophagy in gliomas or GBM [21, 55], which usually manifest as elevated autophagosomes and autophagic flux, mitochondrial matrix swelling or endoplasmic reticulum expansion, increasing expression of LC3, Atg5, Beclin1, and other autophagy-related genes [20, 26, 55]. Kim et al. demonstrate that TTFields induces autophagy in GBM via the AKT2/miR-29b axis [55]. However, autophagy may be a protective mechanism for tumor cells against TTFields [58]. Knockdown of AMPK or ATG7 inhibits TTFields-induced autophagy and results in cell death, suggesting that TTFields-induced autophagy depends on AMPK activation [58]. Colon cancer treated with TTFields alone or in combination with 5-Fluorouracil, or pancreatic cancer treated with TTFields combined with hyperthermia induces autophagy obviously [30, 60].

### Cell cycle arrest

By bioinformatics analysis, TTFields affects mitosis-related processes such as DNA replication and cell cycle [55, 60]. TTFields functionates in the anaphase of mitosis. TTFields prevents cell division by producing heterogeneous intensity at the cleavage furrow of dividing cells, resulting in apocyte [66]. Giladi et al. [35] demonstrate that cell proliferation is inhibited with prolonged exposure to TTFields, and the cells become significantly larger [41]. The rate of metaphase plate formation maintains whether or not TTFields is applied. Meanwhile, with the sustention of mitosis, DNA content heightens after TTFields exposure, which demonstrates that TTFields acts in the anaphase of mitosis [66].

The effect of TTFields depends on the cell cycle. The application of TTFields in the G1 phase does not affect the portion of G1 phase; similarly, the same as in the M phase [66]. For the G1/S phase-blockade cell, TTFields could not induce cell death, apoptosis, and DNA damage [62], indicating that entering into the G1/S phase is necessary for TTFields to inhibit tumors.

The effect of TTFields results in different cell cycle arrest. G2/M arrest often occurs in glioma cells [26, 55, 56]. The changes in the G2/M phase may be related to the duration and frequency of TTFields. Jo et al. [57] explodes the time gradient of TTFields (0, 3, 6, 12, 24 hours/day). With the prolongation of duration, normal cells with G2/M arrest slightly increase, while the increase of glioma cells is significant. However, there is a contradiction in other studies. No difference in the G2 phase is found when TTFields treats for 5 days, while some studies report that the G2 phase increases when TTFields treats at the optimal frequency for 72 hours [45]. G1 and S phases show different trends in various studies [20, 26, 45, 65]. TTFields has no apparent cycle-blocking regularity in other tumors [25, 28, 35, 37, 48].

TTFields leads to apoptosis, the formation of specific-size DNA fragments, which is reflected in the appearance of Sub G1 peak in cell cycle. However, the timing of Sub G1 peak appears inconsistent among cell lines [20, 21, 25]. Lee et al. [68] detect the changes of cycle-related genes after TTFields treatment in cells with different TP53 statuses, which provided research data to elucidate the mechanism.

However, some studies indicate TTFields induces necrosis, immunogenic death, and necroptosis. TTFields does not increase apoptosis at higher intensity and optimal inhibition frequency [20, 63] but induces autophagy and necrosis [20]. Pancreatic cancer cells treated with TTFields for 144 hours show no apoptosis and necrosis but increased apoptosis after TTFields and radiotherapy [50]. TTFields induces ATP release by inducing autophagy, leading to immunogenic death [52]. In addition, the necroptosis induced by TTFields also leads to cell death [20].

In vitro and in vivo studies have shown that TTFields increase cell death through P53-dependent [57], reactive oxygen species elevation [30], caspase-dependent/independent pathways, and O^6^ -methylguanine-DNA methyltransferase (MGMT)-independent pathways [20, 41, 42].

### Permeability (nuclear membrane, cell membrane, blood–brain barrier permeability, anti-angiogenesis) and drug infiltration

TTFields causes local rupture and perforation of the nuclear envelope, which are associated with the cell cycle. Entering into the S phase is required for TTFields to induce nuclear envelope disruption and micronucleus formation [69, 70]. Meanwhile, nuclear membrane disruption, micronuclei formation, and fragmented DNA release after TTFields activate Caspase1 to cleavage GSDMD, which induces pyroptosis and membrane disruption [69, 70].

TTFields enhances cell membrane permeability limitedly, and it is difficult for larger molecular weight substances to penetrate the cell membrane. TTFields causes significant morphological changes in the cytoplasm and membrane, including disruption of plasma membrane integrity and marked vacuolization, with increased membrane permeability [20, 55]. Previous studies have shown that exposure to TTFields at 4 V/cm and 200 kHz for 6-24 hours increases the membrane pores in GBM cells, and the membrane pore area approximately is doubled (240.6 ± 91.7 nm^2^ vs. 129.8 ± 31.9 nm^2^). TTFields only increases the absorption of relatively small molecular weight species such as 4–20 kDa dextran-FITC, 5-aminolevulinic acid, and ethidium D. However, no absorption is observed in relatively larger molecular weight species (≥50 kDa) [63].

The effect of TTFields on membrane permeability is reversible. Twenty-four hours after the termination of TTFields, the number and diameter of membrane pores decrease, and no accumulation of 7-Aminoactinomycin D in cells is observed, indicating that the integrity of cell membrane is repaired [71]. Gera et al. [66] demonstrate that TTFileds resulting in membrane rupture and vacuolization closely related to the timing of cell division, which usually occurs after the formation of mitotic plate.

The frequency of TTFields inducing the permeability of cell membrane or blood-brain barrier was not consistent with the optimal inhibition frequency. Different frequencies (50-500 kHz) of TTFields showed increased intracellular accumulation of 7-Aminoactinomycin D among various tumor cell lines. In all, 100 kHz TTFields changes the permeability of the blood–brain barrier in rats and increases Paclitaxel concentration in GBM. However, in current studies and clinical applications, the optimal frequency for treating GBM is 200 kHz [41, 42, 72].

Although TTFields increases permeability, few researches study the mechanism. TTFields induces the ion channel opening, such as Cav1.2, through cellular depolarization [65, 73]. However, the TTFields frequency of membrane pore opening is inconsistent with the opening of the ion channel. Meanwhile, the pore size that TTFields causes is different from the ion channel opening [63, 74]. Therefore, the opening of the ion channel appears to be secondary. Based on bio-electrorheological models, TTFields-induced changes in membrane shear stress, or electroporation-based models, TTFields-induced changes in the cell membrane and cytoskeleton may further clarify the mechanism of permeability changes [73].

TTFields also opens the blood–brain barrier reversibly, but the relationship with frequency is unclear. The blood-brain barrier is a vital structure to maintain the stability of the internal environment of brain. Chemotherapy for brain tumors usually lacks of effectiveness because most chemotherapeutic drugs are difficult to penetrate the blood-brain barrier [75]. In vivo experiments show that Evans Blue, TRITC-dextran, and magnetic resonance contrast agent Gd-DTPA increase in the brain [75–78]. Meanwhile, in vitro experiments showed that Claudin-5 and Occludin translocation in capillary endothelial cells [76, 79] points out that TTFields increases the permeability of the blood-brain barrier. The current studies have shown that 100 kHz is the best frequency for opening the blood–brain barrier in the rat [75–80]. Permeability is generally most pronounced 24 hours after TTFields exposure [80]. The blood-brain barrier recovery starts 48 hours after termination of TTFields and is fully recovered at 96 hours [75–80].

TTFields exerts antiangiogenic effects and enhances drug penetration. TTFields attenuates tube formation [26] and inhibits angiogenesis by down-regulating the expression of HIF1α, VEGF [47], and MMP2 [81]. In subcutaneous mouse model of melanoma, TTFields reduces the expression of CD34 and VEGF, possibly normalizing vascular and increasing blood flow in solid tumors [53]. Moreover, Kim et al. demonstrate that TTFields facilitates Trastuzumab penetrate to tumors [33].

### Immune modulation

Effects of TTFields on immune cells in vitro. Similar to the pernicious effect of tumor cells, TTFields inhibits the proliferation of RAW264.7 and T cells. However, they also maintain functional activation status (morphological changes, molecular changes such as CD107a, PD-1, and secreted factors such as reactive oxygen species, NO, IL-1β, TNF-α, IFNγ) [82, 83].

Immune activation of TTFields. TTFields not only affects DNA but also alters mitochondrial and endoplasmic reticulum functions, including electron transport, metabolism, ion signaling, and protein folding [68]. After that, TTFields rises to a new mode of cell death. Voloshin et al. [52] demonstrate that TTFields induces immunogenic death of tumor cells (increasing expression of HMGB1, release of ATP, CRT). Hereafter, the product of immunogenic death activate dendritic cells (increased phagocytic index of bone marrow-derived dendritic cells, expression of co-stimulatory molecules such as MHCII, CD40, and CD80) and induce CD45 + leukocyte enrichment.

TTFields increases immune cell infiltration. Although no change is found in peripheral blood WBC, increased CD8 T cells are observed after TTFields treats for 14 days [41]. In the VX2 tumor model, TTFields inhibits the lung metastasis of melanoma. Meanwhile, tumor parenchyma and surrounding tissue are infiltrated with immune cells such as monocytes, CD4, CD8, and CD45 + T cells. Furthermore, among TILs, CD4 T cells are more prevalent than CD8 T cells [84]. Chen et al.’s [69] single-cell sequence results of GBM consistently demonstrate the immune modulation role of TTFields. TTFields increases total and activated DCs (CD80/CD86 + ), early (CD69+) or effector (CD44+/CD62L−) CD4+CD8+.

Possible targets of TTFields modulating immunity. TP53 may be a dependent target of TTFields regulating immune. Among TTFields-induced genes involved in immune and inflammatory responses, TP53-dependent/independent regulated genes were identified by bioinformatics analysis [68]. In addition, RhoA is a crucial factor in regulating leukocyte differentiation and function. Voloshin et al. [51] demonstrate that TTFields markedly and transiently activates RhoA signaling by regulating GEF-H1, resulting in cytoskeletal actin reorganization and focal adhesion formation in lung adenocarcinoma. However, no change in T cells and dendritic cells is observed.

TTFields leads to abnormal micronuclear clusters in GBM, lung adenocarcinoma, and pancreatic cancer cells, which recruits cGAS and AIM2 [69]. Finally, TTFields increases proinflammatory cytokines and type I interferon via the cGAS-STING pathway or the AIM2/caspase1 inflammasome release, resulting in activation of adaptive immunity [70]. Single-cell sequence results indicate that a higher proportion of pDC and T1IRG-expressing monocyte, XCL1/2 + KLRC1 + NK cells, are found in peripheral blood mononuclear cell after TTFields. TTFields promotes T cell activation, memory T cell formation, and peripheral T cell clonal expansion [69].

TTFields usually up-regulates immune checkpoints. Single-cell sequence show that the expression of PD-L1, CTLA-4, and TIGIT increase after TTFields, which provided a theoretical basis for immunotherapy [69]. In addition, TTFields significantly increases CLEC9, IRF8, SMPD3 in cDCs and pDCs, CD8A, IFNG, GZMB, PRF1, CXCR1, CCL4 in TILs [69]. Furthermore, the animal experiment has proven that TTFields combined with anti-PD-1 therapy effectively suppress tumors. However, the molecular mechanism remains unknown [52].

### TTFields combined with radiotherapy

Radiation therapy (RT) causes DNA damage, leading to cell death through apoptosis, mitosis, autophagy, or growth arrest [85]. Regardless of the sequence in which ionizing radiation(IR) and TTFields is applied (TTFields [22, 23] or IR [20, 21, 24, 25] first), most studies show a combined effect. When combined with TTFields, relatively large dose like 4 Gy, 2 Gy are more effective than 2 Gy, 1 Gy [22]. Furthermore, proton therapy is more striking than X-ray [21]. NSCLC cells are more susceptible to radiation when they are exposed to TTFields before IR treatment [22]. The administration of TTFields after 1 h of RT is more pronounced than that after 4 h and 24 h of RT [24]. There are few studies on the combined application of IR and TTFields, mainly focusing on DNA damage and repair [25, 28, 56, 86, 87]. The parameters of TTFields combined with RT to inhibit tumors are shown in Table 2.

**Table 2:** Overview of the frequency, intensity, duration, radiation dose, and dose rate, sequence, and the effect of TTFields combined radiation on various tumor cells.

Cancer type	Cell	Dose and dose rate	TTFields parameters	Sequence	Time and effect	Ref.
Glioma	U-118 MG, LN-18	0–8 Gy (0.25 Gy/min)	200 kHz,1.75 V/cm	RT then TTFields (RT 1 h, 4 h, 24 h then TTFields 72 h)	Radiation sensitization, cell proliferation ↓	[24]
					U-118 MG: γh2AX↑, DNA damage repair↓	
	F98, U373	0-5 Gy X-ray or proton beam (3.45 Gy/min)	150 kHz,0.9 V/cm	RT then TTFields (RT 48 h then TTFields 24 h,48 h)	Radiation sensitization: proton beam> X-rays. Proton beams+TTFields: apoptosis, autophagy↑, migration↓	[21]
	LN-18, LN-229, T-325, ZH-161	3 Gy, 5 Gy	2 V/cm	RT then TTFields	LN-18, T-325: radiation sensitization	[20]
NSCLC	H157, H4006, A549, H1299, H1650	2 Gy, 4 Gy	100–200 kHz	RT then TTFields (RT then TTFields 24 h, 48 h, 72 h)	Radiation sensitization. DNA damage repair↓	[25]
					2 Gy+TTFields 24-72 h:CI:0.58–2.08, among which 53%>1	
					4 Gy+TTFields 24-72 h:CI:0.9–3.97, among which 86%>1.	
	H157, H4006, A549, H1299	2 Gy,4 Gy (3.47 Gy/min)	H157 (100 kHz), H4006(150 kHz),A549(200 kHz), H1299(100 kHz)	TTFields then RT (TTFields 48–72 h then RT)	Radiation sensitization: CI > 1.CI when TTFields first is relatively large	[22]
Pancreatic cancer	CFPAC-I, HPAF-II	5 Gy	150 kHz,0.9 V/cm	TTFields then RT	Radiation sensitization. Clonal formation↓	[23]
					Apoptosis and PARP expression↑	

*↑* up-regulate, *↓* down-regulate, *=* unchanged

*CI* combination index, *RT* radiotherapy, *PARP* poly (ADP-ribose) polymerase, *VS* versus.

### TTFields combined with drugs

Glioma or Glioblastoma. TTFields combined with drugs such as Paclitaxel, Mebendazole [45], Dacarbazine [31], MPS1-IN-3 [49], and Sorafenib [26, 32] significantly increase the sensitivity. MGMT status is often an indication of Temozolomide usage. Experiments on primary cells with different MGMT statuses have shown that Temozolomide and TTFields only have an additive effect (but another cell line with a sensitization phenomenon [20]). However, MGMT status does not affect TTFields efficacy [54]. Additionally, TTFields is synergistic with drugs only within a specific frequency range [36]. Dexamethasone is the most common corticosteroid used to treat edema in GBM patients. Linder et al. demonstrate that Dexamethasone limits radiotherapy efficacy but makes no difference in TTFields-induced GBM cell death. Furthermore, a retrospective analysis shows that Dexamethasone makes no impact in progression-free survival and overall survival when combined with TTFields therapy [44].

Breast cancer. TTFields combined with Doxorubicin, Paclitaxel, or Cyclophosphamide have synergistic effects, manifested as a decrease in half maximal inhibitory concentration (IC50) and dose reduction index (DRI), inhibition of cell proliferation and colony formation, and increased apoptosis [27, 31, 33, 36]. For drug-resistant tumor cells, TTFields combined with drug therapy improves drug resistance [33] and has similar effects on drug-resistant or drug-sensitive cells [27]. Continue propagation after 24 hours treatment of TTFields combined with drug shows that monotherapy group proliferated rapidly, suggesting that combination treatment may have a long-term effect [31].

Lung cancer. TTFields combined with chemotherapeutic drugs such as Cisplatin, Paclitaxel, or Pemetrexed, significantly inhibits proliferation and colony formation [34]. The function of epidermal growth factor receptor (EGRF) inhibitors in combination with TTFields is controversial. Giladi et al. [34] report that Erlotinib combined with TTFields inhibits tumor proliferation, but Li et al. [29] prove that TTFields attenuates the tumor-suppressive effect of Osimertinib. Karanam et al. [22] report that TTFields combined with Olaparib and IR is more inhibitory than the two-factor combination therapy.

Other tumors. MPM [28], abdominal tumors such as colon cancer [30], pancreatic cancer [35], liver cancer [38], ovarian cancer [37], and cervical cancer [38], have reported that TTFields improves the drug’s efficacy. In addition, the function of TTFields combined with hyperthermia is controversial [36, 60, 64]. The parameters of tumor inhibition by TTFields combined with drugs are shown in Table 3.

**Table 3:** Summary of the frequency, intensity, duration, drug concentration, and the effect of TTFields combined drugs on various tumor cells.

Cancer type	Cell	Drug	Concentration	TTFields parameters	Time and effect	Ref.
Ovarian cancer	A2780, OVCAR3, CAOV-3	Paclitaxel	0–100 nM	200 kHz,2.7 V/cm	72 h CI A2780:1.03, OVCAR3:0.81, CAOV-3:0.86.	[37]
MPM	MSTO-211H, NCI-H2052	Cisplatin	1–10,000 nM	150 kHz, 1 V/cm	Pemetrexed+TTFields vs. Cisplatin+TTFields: additive vs. synergistical effect.	[28]
		Pemetrexed	1–100 nM		Cisplatin+TTFields: Apoptosis↑	
					Triple therapy (two drugs+TTFields): proliferation and clonal formation↓	
Lung cancer	Gefitinib-resistant PC-9GR, H1975 cells	Osimertinib	0.5 μM	1 V/cm	Proliferation↑, cell death, apoptosis↓	[29]
					TTFields attenuates the inhibitory effect of Osimertinib	
	HCC827	Erlotinib	0–20 nM	150 kHz,1.75 V/cm	72 h: Proliferation and clonal formation↓	[34]
	H1299, LLC1, HTB-182, KLN205	Pemetrexed	0–0.1 nM	150 kHz,1.75 V/cm	72 h: Proliferation and clonal formation↓	[34]
		Paclitaxel	0–100 nM			
		Cisplatin	0–10 nM			
	H157, H4006, A549, H1299	Cisplatin	Cisplatin PIC25 (μM) : H157 1, H4006 0.75, A549 2, H1299 = 2.	100-200 kHz	Olaparib+IR + TTFields:CI>1.	[22]
		Olaparib	Olaparib 0–40 μM		Olaparib+IR, Olaparib+TTFields:CI ≈ 1.	
Liver cancer	Huh7	Doxorubicin	0–10 µM	120 kHz, 1 Vpp	72 h: therapeutic effect↑	[38]
Glioma	LN-18, LN-229, T-325, ZH-161	TMZ	5 μM-200 μM	2 V/cm	24 h: LN-229, ZH-161: sensitization	[20]
	U118	Dacarbazine	0-100 mM	150 kHz, 1.75 V/cm	72 h: IC50: Dacarbazine 6.4 mM→0.023 mM, Paclitaxel 5 nM→0.005 nM, Doxorubicin 0.04 μM → 0.002 μM, Cyclophosphamide 6.6 mM→0.044 mM.	[31]
					DRI: Dacarbazine 175, Paclitaxel 316, Doxorubicin 23, Cyclophosphamide 152	
	U373	Thymidine	2 mM	200 kHz	For G1/S blocked cells, proliferation, clonal formation, DNA damage and apoptosis=	[62]
	U373, U87	Sorafenib	5 μM	150 kHz, 0.9 V/cm	48 h: proliferation ↓ , cell death ↑ .	[26, 32]
					STAT3 expression ↓ . Knocking down STAT3: TTFields effect↑	
					24 h: S phase, migration, invasion and angiogenesis↓	
					48 h: clonal formation ↓ , apoptosis, autophagy, ROS ↑	
	U-87 MG, U-138 MG, U-343 MG	MPS1-IN-3	4 µM	200 kHz, 1.7 V/cm	U-87 MG, GaMG: 72 h:proliferation ↓ .	[49]
					U-87 MG, 72 h: abnormal nuclei, G2/M, apoptosis ↑ .	
					MPS1-IN-3 prolongs TTFields effect	
	U87-MG, KNS42, SF188	Paclitaxel	High vs Low concentration	130 kHz, 10 V, 450 μs	Synergistically effect.	[45]
		Mebendazole			SubG0↑	
GBM	KR158-luc	TMZ	300 µM	200 kHz	72 h: adaptive immune=	[69]
	Patient-derived glioblastoma stem cell-like cells (GSCs)	TMZ	1.5 μM-160 μM	200 kHz, 1 V/cm	8 days: same inhibitory efficacy in two cell types (MGMT expression + /-).	[54]
	U251-MG, MZ-54	Dexamethasone	65 μM	1.48–1.41 V/cm	48 h: sensitization.	[44]
					Dexamethasone: decrease IR induced-effect but does not affect TTFields induced-effect	
					Dexamethasone+TTFields: PFS and OS =	
	U87-MG, GBM2, GBM39	Withaferin A	0–0.1uM	200 kHz, 4 V/cm 2.5 V/cm	50 kHz vs. 200 kHz, 500 kHz: no sensitization vs. sensitization. Intensity dependent	[36]
Colonic cancer	HCT116	5-FU	5 µmol/L	0.9–1.2 V/cm	48 h: sensitization.	[30]
					Proliferation, clonal formation, migration, invasion↓	
					Autophagy, apoptosis, organoid cell death↑	
Cervical cancer	HeLa	Doxorubicin	0–10 µM	120 kHz, 1 Vpp	72 h: therapeutic effect↑	[38]
Breast cancer	Emt^R1^ cells, MCF-7/Mx, MDA-MB-231/Dox cells	Doxorubicin	0.04–0.6 μM,	150 kHz, 1.75 V/cm	TTFields+chemotherapy 72 h: Same efficacy in WT cell and drug-resistant cell. DRI: Doxorubicin 105-250 vs. Paclitaxel 815- >10,000	[27]
		Paclitaxel	5 nM–0.1 μM			
	JIMT-1,BT-474	Trastuzumab	5 μM	–	72 h: Synergistical effect, clonal formation ↓ , apoptosis↑	[33]
	MDA-MB-231	Doxorubicin	IC50 = 0.31 μM	150 kHz, 4 V/cm	Synergistical effect	[36]
	MDA-MB-231	Doxorubicin, Paclitaxel, Cyclophosphamide	0–10 μM, 0–1000 nM, 0–100 mM	150 kHz, 1.75 V/cm	72 h IC50: Doxorubicin 0.04 μM → 0.002 μM, Paclitaxel 5.00 nM→0.005 nM, Cyclophosphamide 6.60 mM→0.044 mM.	[31]
					24 h treatment then quit for 48 h: Control and the chemotherapy group vs. Combined group: cell proliferation recovered rapidly vs. did not recover.	
Pancreatic cancer	PC-1.0 (hamster), AsPC-1	Gemcitabine	–	150 kHz, 2.9 ± 0.2 V/cm	Therapeutic effect↑	[35]
		Irinotecan				
		5-FU				
		Paclitaxel				
Liver cancer	Huh7	Doxorubicin	0–10 µM	120 kHz, 1 Vpp	72 h: therapeutic effect↑	[38]

*↑* up-regulate,*↓* down-regulate, *=* unchanged.

*5-FU* 5-Fluorouracil, *WT* wide type, *DRI* dose reduction index, *IC50* half maximal inhibitory concentration, *CI* combination index, *TMZ* Temozolomide, *STAT3* signal transducer and activator of transcription 3, *MGMT* O6 -methylguanine-DNA methyltransferase, *OS* overall survival, *PFS* progression-free survival, *MDA-MB-231/Dox cells* doxorubicin resistant MDA-MB-231 cells, *EmtR1 cells* AA8 cells- Emetine-resistant sub-lines, *MCF-7/Mx* MCF-7 cells Mitoxantrone-resistant sub-lines, *Vs* versus.

### Molecular mechanism

**Fig. 1:**
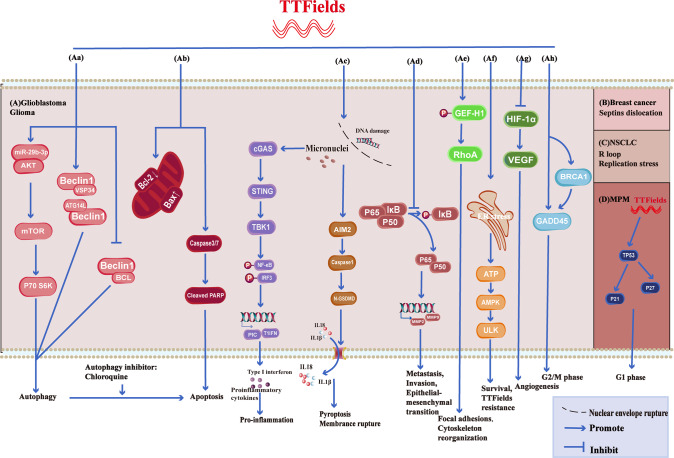
Molecular pathway changes caused by TTFields on glioma, GBM, MPM, NSCLC, and breast cancer Fig. 1A, Gliomas and glioblastomas. **A** Aa After TTFields treatment, Beclin1 increases the binding of Atg14L and Vps34(the positively regulated autophagosome) and decreases Bcl-2(the negatively regulated autophagosome), leading to glioma cells and tumor stem cell autophagy. Meanwhile, activation of the AKT2/mTOR/p70S6K axis also leads to autophagy. **A** Ab TTFields up-regulates caspase3, caspase7 or increases BAX, down-regulates BCL-2 expression, and leads to apoptosis. **A** Ac TTFields destroys the nuclear membrane, generates micronuclei and double strand breaks, activate the cGAS-Sting signaling pathway to increase the expression of proinflammatory factors and type I interferon, and through the AIM2-Caspase1 inflammasome Cleavage of GSDMD and release of LDH leads to pyroptosis and immune activation ultimately. **A** Ad TTFields inhibits IκBα phosphorylation and NF-κB p65 translocation, the expression of MMP2 and MMP9, and ultimately inhibits cell invasion, metastasis, and EMT processes. **A** Ae TTFields promotes phosphorylation of GEF-H1, which further activates RhoA, ultimately leading to focal adhesions and cytoskeleton reorganization. **A** Af TTFields causes Endoplasmic Reticulum stress and releases ATP, which activates AMPK and ULK, leading to resistance to TTFields. **A** Ag TTFields attenuates tube formation and angiogenesis by down-regulating the expression of HIF1α and VEGF. **A** Ah Upregulation of BRCA1 and GADD45 results in G2/M phase arrest. **B** Breast cancer. Septins are abnormally distributed. **C** Non-small cell lung cancer. TTFields lead to R loop formation and replication stress. **D** MPM. Elevated TP53, P21, and P27 lead to G1 phase blockade.

TTFields leads to abnormal Septins distribution and tubulin assembly blockage (Fig. 1B). Septins are integral components of the cytoskeleton, assembling into higher-order oligomers and filamentous polymers associated with actin filaments, microtubules, and cell membranes. Thus, abnormally expressed Septins may destabilize genomes [88]. Gera et al. [66] find that after TTFields treatment, the localization of Septins in the midline of the anaphase spindle and cell kinetic division grooves is significantly reduced and disorganized, resulting in abnormal progeny cells. Meanwhile, the mitotic spindle is abnormal in metaphase and telophase, resulting in the formation of abnormal cells, such as apocyte and abnormal chromosome, and the number of cells in interphase and telophase is reduced [35, 48, 56]. In addition, Voloshin, T et al. [51] demonstrate that despite blocking the assembly of tubulin proteins, TTFileds affects the directionality and cell polarity of tubulin. The small GTPase RhoA regulates stress fiber assembly and focal adhesion formation [89–91]. Disruption of tubulin after TTFields activates RhoA signaling by modulating GEF-H1 phosphorylation, leading to cytoskeletal actin reorganization and formation of focal adhesions (Fig. 1A, Ae) [48, 51].

Based on bioinformatics analysis, numerous gene expression and pathway changes are found, which is conducive to the in-depth study of TTFields. The PI3K-AKT, MAPK, DNA replication, cell cycle, and other pathways have been confirmed.

TTFields slows down replication forks and caused replication stress (Fig. 1C). After 72 hours of TTFields treatment, RPA increases and DNA fiber length decreases. TTFields induces replication stress with reducing genes expression of key regulators in mitotic and replication stress [22]. The nascent RNA binds to the template DNA strand during transcription, forming a unique RNA-DNA hybrid structure named the R-loop [92]. TTFields increased R-loop formation.

**Fig. 2:**
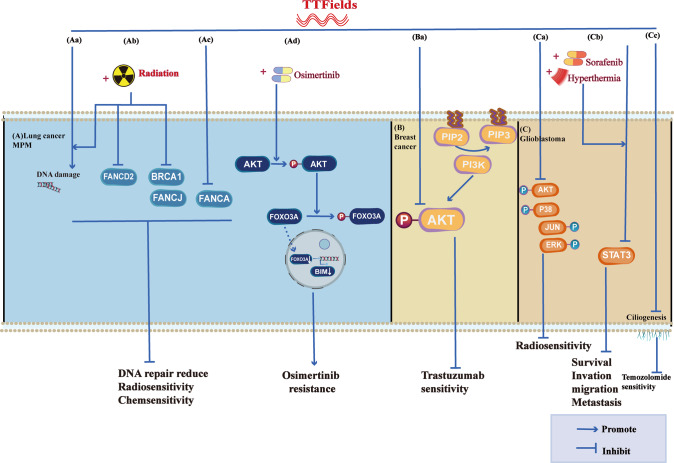
Molecular pathway changes caused by TTFields combined with radiotherapy or drugs in GBM, MPM, NSCLC, and breast cancer. **A** Lung cancer or MPM. **A** Aa–c TTFields combined with radiation causes DNA damage but reduces DNA damage repair by inhibiting the expression of FANCA, FANCD2, FANCJ, and BRCA. **A** Ad In addition, TTFields promotes the phosphorylation of AKT, which in turn promotes the phosphorylation of FOXO3A, reduces the nuclear entry of FOXO3A, and inhibits the expression of BIM, which ultimately leads to the weakening of the efficacy of Osimertinib. **B** Breast cancer. **B** Ba TTFields enhances breast cancer sensitivity to Trastuzumab by inhibiting AKT phosphorylation. **C** Glioblastoma. **C** Ca TTFields inhibits the phosphorylation of AKT, JUN, P38, and ERK, resulting in enhanced radiosensitivity while inhibiting ciliogenesis and enhancing the sensitivity of GBM to Temozolomide. **C** Cb In addition, TTFields combined with Sorafenib or hyperthermia resulted in cell death by inhibiting STAT3. **C** Cc TTFields inhibits ciliogenesis, thereby suppressing sensitivity to Temozolomide.

NF-κB, PI3K/AKT, and MAPK signaling pathways. TTFields inhibits IκBα phosphorylation and NF-κB p65 translocation, which suppresses MMP2 and MMP9 by downregulating NF-κB signaling [47] or inhibits GBM invasion and migration through epithelial-mesenchymal transition (EMT) (Fig. 1A, Ad) [47, 81]. The PI3K/AKT/mTOR signaling pathway is involved in the growth and survival of various tumors [93]. Targeting PI3K/AKT/mTOR-mediated autophagy is not only an essential strategy for treating tumors but also plays a vital role in improving the sensitivity of tumor cells to radiotherapy and chemotherapy. TTFields attenuates the efficacy of Osimertinib by activating p-AKT and p-FOXO3a and inhibiting the nuclear translocation of FOXO3a (Fig. 2A, Ad) [29]. However, other studies have shown that TTFields improves breast cancer sensitivity to Trastuzumab (Fig. 2B, Ba) and GBM radiosensitivity by downregulating p38, p-JNK, p-AKT, p-ERK, and p-HER2 (Fig. 2C, Ca) [21, 33]. In addition, TTFields activates RAW 264.7 cells by activating MAPK and NF-kB signaling pathways [83]. In addition, Shteingauz et al. [58] prove that TTFields induces autophagy by activating ULK1 in an AMPK-dependent manner, resulting in TTFields resistance (Fig. 1A. Af).

TTFields influences the expression of AKT2, a critical targets for regulating autophagy. Kim et al. [55] report that after TTFields, Beclin1-Atg14L/Vps34 complex increases and Beclin1-Bcl-2 complex decreases in glioma cells and tumor stem cell, leading to autophagy through AKT2/mTOR/p70S6K axis. Meanwhile, TTFields up-regulates miR-29b-3p, targeted binding AKT2, resulting in the decreased expression of AKT2 (Fig. 1A, Aa).

STAT3, a cytoplasmic transcription factor and a downstream molecule of mTOR, is activated in various cancers, including hematological malignancies and solid tumors, to induce proliferation, invasion, metastasis, and angiogenesis [94, 95]. TTFields alone, combined with Sorafenib or hyperthermia, downregulates STAT3 in GBM, resulting in enhanced efficacy (Fig. 2C, Cb) [32, 64].

TTFields induces type I interferon and proinflammatory cytokines via the cGAS-STING pathway, which may lead to immune activation. The cGAS-STING pathway is involved in pyroptosis. In case of infection, cellular stress, and tissue damage, the cGAS-STING signaling pathway senses DNA damage and regulates infection, inflammatory diseases, and tumor immunity [96–98]. TTFields alone or in combination with radiotherapy destroy the nuclear membrane, and generate micronuclei and double strand breaks of DNA, which activates the cGAS-STING signaling pathway to increase the expression of proinflammatory factors and type I interferon [28]. Meanwhile, TTFields leads to pyroptosis and immune activation via the AIM2-Caspase1 inflammasome which slices GSDMD and releases LDH (Fig. 1A, Ac) [69].

TTFields regulates DNA damage repair, radiation and drug resistance via the Fanconi Anemia-BRCA pathway. Genomic instability is often associated with tumorigenesis, and the Fanconi Anemia-BRCA pathway is involved in the repair of interstrand crosslinks and double-strand DNA breaks by homologous recombination [86, 87]. The effect of TTFields on BCRA1 expression is controversial. Jeong et al. [56] show that TTFields increases the expression of BRCA1, GADD45, TP53, and FOXO3A, and decreased protein expression of CDC2 and Cyclin B1, respectively, confirming the occurrence of G2/M phase arrest (Fig. 1A, Ah). However, TTFields combined with IR down-regulates BRCA1 expression and reduces DNA double-strand breaks repair, resulting in sensitivity to ionizing radiation (Fig. 2A, Ab) [25]. Likewise, TTFields down-regulates the Fanconi Anemia-BRCA pathway, promoting chemosensitivity in malignant pleural mesothelioma (Fig. 2A, Ac).

In addition, p21 and p27 are elevated after TTFields, which activates the cell cycle checkpoint (Fig. 1D) [28, 56]. TTFields also inhibits angiogenesis by suppressing HIF1α and VEGF (Fig. 1A, Ag) [47]. TTFields down-regulates BCL2, up-regulates cleaved PARP and BAX [33], and induces apoptosis in breast cancer, ovarian cancer, and glioma cells through a caspase-dependent pathway (Fig. 1A, Ab) [48].

Other mechanisms show that TTFields activates the Cav1.2 ion channel resulting in permeability [65], and inhibits ciliogenesis thereby enhancing Temozolomide toxicity (Fig. 2C. Cc) [99]. The molecular pathway changes of TTFields alone or in combination with other treatments are shown in Figs. 1 and 2.

### Conclusion and future perspectives

TTFields is a non-invasive tumor therapy. In vitro/vivo experiments and clinical trials have demonstrated the therapeutic effects of TTFields alone or in combination with radiotherapy and chemotherapy in various tumors [8, 9, 15, 17, 19, 24, 31]. For the first time, we summarize the relevant parameters of TTFields used in the current studies, such as frequency, intensity, duration. TTFields changes the effect of radiotherapy [20–25] and chemotherapy [26–38]. Therefore, we summarize the combined regimen of current researches, but it is difficult to make clear the best combination scheme due to the lack of adequate research. Last but not least, we firstly sum up the pathway and molecular alternation of TTFields.

**Fig. 3:**
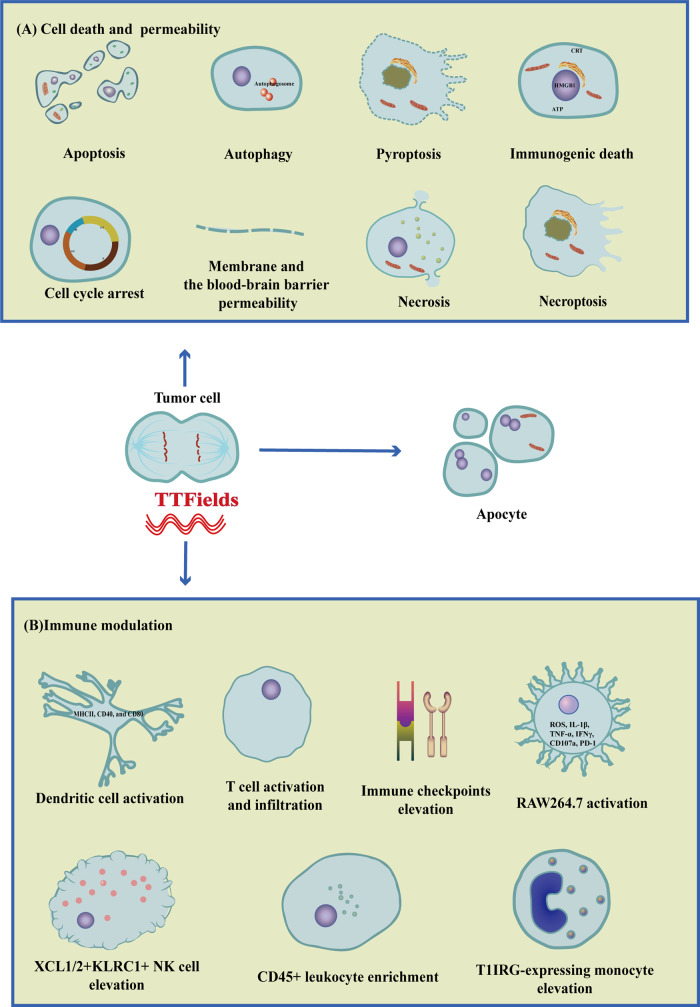
TTFields induces cell death, permeability and immune modulation. The classical effect of TTFields is mitosis inhibitions and formation of apocyte. **A** TTFields induces various mode of tumor cell death, including apoptosis, autophagy, pyroptosis, immunogenic death, necrosis, necroptosis, and cell cycle arrest. Meanwhile, TTFields affects the integrity of membrane and the blood-brain barrier, increasing permeability of tumor cell. **B** TTFields induces activation of dendritic cell, RAW264.7. In addition, TTFields leads to T cell infiltration and CD45 + leukocyte enrichment. Meanwhile, T1IRG-expressing monocyte, NK cell and immune checkpoints are elevated after TTFields treatment.

TTFields studies are relatively limited, but the future is bright. First, we should consider parameters related to TTFields. More studies should focus on combining TTFields and radiotherapy or chemotherapy, making clear the best-combined formula [20–38]. In addition, TTFields promotes various mode of cell death (Fig. 3A). TTFields induces the immunogenic death of tumor cells, releases proinflammatory factors, activates immune cells, and adaptive immunity [69]. As a non-invasive physical therapy, TTFields plays an essential role in regulating immune function (Fig. 3B). Its combination with anti-PD-1 significantly inhibits tumors [52], which attracts us to pay more attention to the combination of TTFields and immunotherapy.

At present, although a small part of fundamental researches study on various tumors, such as lung cancer [25, 34, 48, 52, 58], breast cancer [1, 27, 31, 36, 48], and pancreatic cancer [50], it mainly focuses on glioma [1, 20, 57] and GBM [1, 36, 44, 45, 47–49, 51, 54–56, 63]. In addition, with the emergence of drug or radiotherapy resistance, the role of TTFields is not yet conclusive. We look forward to applying TTFields in other tumor cells or drug-/radio-resistant cell lines and clarifying its mechanism and changes in molecular pathways.

The anti-mitotic effect of TTFields is first discovered to inhibit tumor cells’ division and proliferation. And the current researches have undoubtedly proven that TTFields induces apoptosis [26, 32, 45, 49, 55, 57, 64, 65] and autophagy [20, 21, 26, 30, 55, 58, 60] in cells, leads to cell membrane permeability [20, 55, 63, 66, 69–71], immune regulation [41, 51, 52, 69, 70, 82, 83], resulting in tumor cell killing. In molecular pathway research, TTFields inhibits tubulin assembly and direction, and changes the distribution of Septins [51, 66]. Thus, DNA replication stress and damage increases, activating DNA damage-related pathways, such as the cGAS-STING pathway [69] and Fanconi anemia-BRCA pathway [25, 56]. In addition, TTFields inhibits cell proliferation and promotes sensitivity to radiation or drugs through NF-κB, MAPK, and PI3K/AKT signaling pathways [21, 29, 33, 47, 55, 58, 81, 83, 93]. We look forward to bioinformatics analysis such as single-cell sequence to discover new molecular mechanisms.

## Data Availability

Data openly available in a public repository.
